# Toward a phenomic analysis of chronic postsurgical pain following cardiac surgery

**DOI:** 10.1080/24740527.2019.1580142

**Published:** 2019-04-12

**Authors:** Hance Clarke, Ajit Rai, James Bao, Michael Poon, Vivek Rao, George Djaiani, Scott Beattie, Gabrielle Page, Manon Choiniere, Michael McGillion, Monica Parry, Judith Hunter, Judy Watt-Watson, Loren Martin, Liza Grosman-Rimon, Dinesh Kumbhare, John Hanlon, Ze’ev Seltzer, Joel Katz

**Affiliations:** a Department of Anaesthesia and Pain Management, Toronto General Hospital, Toronto, Ontario, Canada; b Transitional Pain Service, Department of Anaesthesia and Pain Management, Toronto General Hospital, Toronto, Ontario, Canada; c Department of Anaesthesia, University of Toronto, Toronto, Ontario, Canada; d University of Toronto Centre for the Study of Pain, University of Toronto, Toronto, Ontario, Canada; e Department of Cardiovascular Surgery, University Health Network, Toronto General Hospital, Toronto, Ontario, Canada; f Département d'anesthésiologie et médecine de la douleur, Université de Montréal, Montreal, Quebec, Canada; g School of Nursing, McMaster University, Hamilton, Ontario, Canada; h Lawrence S. Bloomberg, Faculty of Nursing, University of Toronto, Toronto, Ontario, Canada; i Department of Physical Therapy, University of Toronto, Toronto, Ontario, Canada; j Department of Psychology, University of Toronto Mississauga, Toronto, Ontario, Canada; k Department of Physical Medicine and Rehabilitation, University of Toronto, Toronto, Ontario, Canada; l Department of Anaesthesia, St. Michael’s Hospital, Toronto, Ontario, Canada; m University of Toronto Faculties of Dentistry and Medicine, University of Toronto, Toronto, Ontario, Canada; n Central Institute of Mental Health, University of Heidelberg, Mannheim, Germany; o Department of Psychology, York University, Toronto, Ontario, Canada

**Keywords:** chronic postsurgical pain, postoperative pain, anxiety, pain catastrophizing, quantitative sensory testing

## Abstract

**Background**: Despite the same surgical approach, up to 40% of patients develop chronic postsurgical pain (CPSP) following cardiac surgery, whereas the rest are chronic pain free. This variability suggests that CPSP is controlled partially through genetics, but the genes for CPSP are largely unknown.

**Aims**: The aim of this study was to identify potential CPSP phenotypes by comparing patients who developed CPSP following cardiac surgery vs. those who did not.

**Methods**: A research ethics board–approved, cross-sectional study of post–cardiac surgery pain was conducted at Toronto General Hospital from 2011 to 2015. Patients were recruited to complete a short survey of chronic pain scores and the Short-Form McGill Pain Questionnaire–2. A subset of patients completed a longer survey of eight validated pain phenotyping questionnaires and/or four psychophysical assessments. All surveys and psychophysical testing were conducted after surgery. Patients were stratified by presence of chronic pain and groups were compared using descriptive statistics.

**Results**: Six hundred forty-three patients completed the short form survey. The mean postsurgery assessment time was 41.5 (SD = ±25.1) months. Over a quarter (27.8%) reported CPSP at the chest as a consequence of their surgery. Of patients reporting CPSP, 46.6% reported mild pain (0–3), 35.8% reported moderate pain (4–7), and 17.6% reported severe pain (7–10) in accordance with the numerical rating scale. Patients with moderate and/or severe CPSP were younger, had a greater body mass index, and had higher anxiety sensitivity, pain catastrophizing, and somatization scores.

**Conclusions**: Chronic pain levels after cardiac surgery are associated with anxiety, catastrophizing, and sensory abnormalities in body parts outside the field innervated by injured nerves, indicating the presence of widespread central sensitization to incoming sensory inputs from intact nerves.

## RÉSUMÉ

**Contexte**: Malgré qu’ils aient été soumis à la même approche chirurgicale, jusqu’à 40 % des patients souffrent de douleur chronique postopératoire après une chirurgie cardiaque, tandis que le reste des patients n’en souffrent pas. Cette variabilité porte à croire que la douleur chronique postopératoire est en partie maitrisée génétiquement, mais les gènes en cause dans la douleur chronique postopératoire sont très peu connus.

**But**: Identifier les phénotypes de douleur chronique postopératoire possibles en comparant des patients souffrant de douleur chronique postopératoire à des patients n’en souffrant pas après une chirurgie cardiaque.

**Méthodes**: Une étude transversale de la douleur après une chirurgie cardiaque approuvée par la commission d’éthique de la recherche a été menée à l’Hôpital général de Toronto de 2011 à 2015. Les patients ont été recrutés pour répondre à un court questionnaire portant sur les scores de douleur chronique et à une version abrégée du McGill Pain Questionnaire-2. Un sous-ensemble de patients a répondu à une enquête plus longue comprenant huit questionnaires validés portant sur le phénotypage de la douleur et/ou sur quatre mesures psychophysiques. Tous les questionnaires et les tests psychophysiques ont été menés après la chirurgie. Les patients ont été stratrifiés en fonction de la présence de douleur chronique et les groupes ont été comparés à l’aide de statistiques descriptives.

**Résultats**: 634 patients ont répondu à la version courte de l’enquête. Le temps moyen de l’évaluation post-chirurgie était de 41,4 mois (écart-type ± 25,1). Plus d’un quart (27,8%) des participants ont rapporté de la douleur chronique postopératoire au thorax en tant que conséquence de la chirurgie. Parmi les patients rapportant de la douleur chronique post-opératoire, 46,6 % ont rapporée une douleur faible (0-3), 35,8 % ont rapporté de la douleur modérée (4-7) et 17,6 % ont rapporté de la douleur sévère (7-10), selon l’échelle d’évaluation numérique. Les patients souffrant de douleur chronique postopératoire de modérée à sévère étaient plus jeunes, avaient un indice de masse corporelle plus élevé et obtenaient des scores plus élevés en ce qui concerne la sensibilité à l’anxiété, la catastrophisation de la douleur et la somatisation.

**Conclusion**: Les niveaux de douleur chronique après une chirurgie cardiaque sont associés à l’anxiété, à la catastrophisation et à des anomalies sensorielles dans des parties du corps à l’extérieur de la zone innervée par les nerfs par les nerfs endommagés, ce qui indique la présence d’une sensibilisation centrale généralisée aux signaux sensoriels provenant des nerfs intacts.

## Introduction

Chronic pain has been coined the silent epidemic of our times, affecting as many as 29% of adults.^1^ Chronic pain is a complex disease that drastically impairs quality of life and is often refractory to available treatments.^2^ It manifests in an aversive–affective dimension with a valence of unpleasantness and suffering to the pain but also has cognitive–evaluative attributes that relate to its global meaning and the context in which it occurs. These dimensions are discrete entities that can be phenotyped with psychometrically sound instruments.^3^

Recently, there has been hope that a pain genetics approach may identify new pharmacogenetic targets allowing for development of novel pre-emptive analgesics. This would allow for individualized preventive and palliative treatments that could reduce the number of individuals transitioning to chronicity and alleviate pain in those who have already developed it.^4–7^

The development of chronic postsurgical pain (CPSP) as a consequence of a midline sternotomy following cardiac surgery is a valuable model for genome-wide association studies for chronic pain. The time of the operation is precisely known, and the extent of nerve injury is fairly consistent given the same surgical approach (harvesting the left internal mammary artery or bilateral internal mammary arteries and/or the saphenous vein). Moreover, the surgical approach and postoperative analgesic care are standardized within institutions. In addition, CPSP following cardiac surgery presents with distinct neuropathic pain traits that are easily differentiated from preoperative angina pectoris pain that might linger postoperatively.

Prospective studies of chronic pain after cardiac surgery indicate a 1-year incidence ranging from 11% to 40%.^8^ Patients undergoing coronary artery bypass graft surgery (CABGS) generally report a significant decline in poststernotomy pain up to 6 months postoperatively. After this period, the pain intensity stabilizes at a level where it remains for years thereafter.^9,10^ In one of the largest prospective studies of 1400 patients with chronic post-CABGS pain, Choinière et al. found the incidence of chronic pain post-CABGS to be 40% at 3 months, 22% at 6 months and 17% at 12 months.^8^ This study identified numerous risk factors for pain post-CABGS, including female gender, younger age, preoperative anxiety, pain catastrophizing and angina, intra-operative opiate use, intense postoperative pain, greater postoperative complications, and worst pain intensity during hospitalization.^8^

In the present study, we sought to delineate phenotypic differences among patients who developed CPSP following cardiac surgery vs. those who did not using (1) validated pain and psychological questionnaires and (2) psychophysical tests. The results are expected to identify robust intermediate phenotypes for genetic analysis to identify candidate genes as targets of novel preventative and palliative care of chronic postsurgical pain.

## Methods

### Study participant overview

A cross-sectional study of post–cardiac surgery pain was approved by the research ethics boards at the Toronto General Hospital (TGH) and York University (Research Ethics Board: 10-0852-AE). This study was conducted at TGH from 2011 to 2015. Patients who (1) previously had cardiac surgery at TGH and (2) gave consent for future contact with regard to their postsurgical recovery and novel studies were eligible to participate. Those who consented and gave a previous blood sample to our Genetic Biobank were contacted by telephone by a member of our team at least 3 months after their cardiac surgery. This cross-sectional survey of pain and psychological phenotypes included three separate pain assessments: a short survey, a long survey, and a psychophysical assessment. The goals and study procedures were explained as part of informed consent. Patients had the option to participate in solely the short survey, both short and long surveys, or all three assessments. Patients also consented to use of their blood samples in a future genomic study.

Prior to surgery, all patients received standardized peri-operative care. Electronic health records were used to collect patient details, including surgical technique (graft sites, length of procedure), intra-operative anesthetic variables (hemodynamic parameters, opiate consumption, use of amnestic agents, transfusion records, cardiopulmonary bypass time), cardiovascular intensive care unit postoperative documentation (hemodynamic values, opiate consumption, length of hospitalization stay), and hospital discharge date. Participants were phenotyped for chronic postsurgical pain and some additionally completed a series of psychophysical tests. All surveys (short and long) and psychophysical testing were conducted after surgery.

### Short survey

For patients participating in the short survey alone, verbal consent was obtained and the consent form was sent by mail with a stamped return envelope. Once the consent form was returned, the patient was sent the survey questionnaires and a stamped return envelope. Participants were free to fill out the questionnaires independently or with the assistance of an interviewer via telephone. The short survey utilized numeric rating scales (NRSs) to determine postsurgical pain levels. NRSs provide patients with chronic pain sufficient discriminative power to describe typical pain episode intensity ranging from 0 to 10: mild (0–3), moderate (4–7), or severe (8–10).^11^ The McGill Pain Questionnaire–Short Form–2 (SF-MPQ-2) also assessed the quality and intensity of specific pain descriptors.^12^ The short survey included details to distinguish differences between CPSP, pain that existed pre-surgery and spontaneous pain. In cases where the postsurgical pain was episodic, we collected data on the intensity, unpleasantness, duration, and frequency of a typical pain episode.

### Long survey

For patients participating in the long survey, consent was obtained and data were collected in a manner identical to that for the short survey. The long survey consisted of eight validated questionnaires to assess psychosocial factors relevant to the development of chronic postsurgical pain. These included the Anxiety Sensitivity Index (ASI-3), the Pain Catastrophizing Scale (PCS), the Pain Anxiety Symptoms Scale–20 (PASS-20), the Posttraumatic Stress Disorder Checklist–Civilian Version (PCL-C), the Hospital Anxiety and Depression Scale (HADS), the Symptom Checklist (SCL)-90-R–Somatization, Mindful Attention Awareness Scale (MAAS), and the Sensitivity to Pain Traumatization Scale (SPTS).

### Psychophysical assessment

The laboratory invited patients who consented to undergo psychophysical assessment to the laboratory at TGH. The series of tests performed included the following.

#### Mechanical pressure pain threshold

Pain thresholds in response to mechanical pressure applied to the skin were obtained using a pressure algometer (Wagner Instruments, Greenwich, CT) that registers the applied force. Pressure pain threshold (PPT) was determined as the force (pressure/unit area, lb/in^2^) at which the patient first reported pain (using an NRS_0–10_). PPTs were obtained 2.5 cm away from the surgical scars on the mid-sternum, at the saphenous vein graft, and at control sites on the right and left forearms in the anterior aspect midway between the wrist and elbow. The pressure algometer has been used to obtain PPTs in previous studies.^13^

#### Thermal pain and sensory thresholds

Thermal stimuli were delivered using the Medoc Pathway pain and sensory evaluation system (Ramat Yishai, Israel). A thermode (16 mm by 16 mm) was applied to the skin on the ventral aspect of the subject’s right forearm and 2.5 cm to the right of the surgical scar on the mid-sternum. When activated, the temperature of the thermode rose or decreased from a baseline temperature of 32°C at a rate of 1°C per second; the patients first detected the temperature as warm or cool, hot or cold, or painfully hot or painfully cold. These sensory detections were made by the patient by depressing a mouse button, which returned the thermode to the baseline temperature at a rate of 1°C per second. Heat and cold pain detection thresholds and thermal detection thresholds to warm and cool stimuli were obtained using the method of limits.^14^

#### Vasoconstrictor inspiratory gasp

This test is known to strongly activate the sympathetic system, which may aggravate neuropathic pain in some patients with chronic pain suffering from sympathetically maintained pain.^15^ Participants rested supine on a couch and relaxed with eyes closed and were asked to take the deepest possible inspiration and then breathe normally. Ratings of ongoing spontaneous pain were taken at 1 min after expiration. Two baseline blood pressure (systolic, diastolic, and mean arterial pressure) and pulse rate measurements were taken just before the gasp test and at 1 min thereafter, just after the patient reported his or her pain ratings.

#### Cold pressor pain test

Participants immersed the right arm just above the elbow in a coldwater/ice bath (1°C) until they could not tolerate the pain any longer and withdrew at their pain tolerance threshold.^16^ This threshold was recorded by the experimenter with a stopwatch as the time from immersion to withdrawal, with a cutoff at 3 min. Participants then used the NRS_0–10_ to rate the pain intensity and unpleasantness of each stimulus. Changes in cardiovascular nociceptive reflexes caused by the cold pressor pain test were recorded as well and compared to the respective baseline values recorded prior to the cold pressor pain test.

### Statistical analysis

Survey data were entered from collected surveys into a Microsoft Excel database and analysis was conducted using IBM SPSS Statistics for Windows, version 25 (IBM Corp., Armonk, NY). Patients were stratified by their chronic pain status (no, mild, moderate, and severe CPSP) and analyzed using descriptive and inferential statistics. For the short survey, long survey, and psychophysical assessments, univariate general linear models were used to identify variables associated with CPSP status. As a second step, three multivariable multinomial regression models using a backward selection procedure were used to identify factors associated with CPSP status; models were run separately for the short survey, long survey, and psychophysiological data. Age and sex were always forced into the regression models. For the short survey data, variables entered in the multivariable model included body mass index (BMI), preoperative chronic pain status, time from surgery to study consent, and total score on the SF-MPQ-2. For the long survey data, variables entered included BMI, preoperative chronic pain status, and total scores on measures of anxiety sensitivity (ASI-3), anxiety and depression (HADS-D and HADS-A), pain anxiety (PASS-20), sensitivity to pain traumatization (SPTS), posttraumatic stress symptoms (PCL-C), pain catastrophizing (PCS), mindful awareness (MAAS), and somatization (SCL-90-R). As for the psychophysiological data, variables entered included pain scores on the different tests for which sufficient data were collected in each CPSP status category. These variables included mechanical pressure pain threshold–mid-sternal scar, cold pressure pain test 30 s postwithdrawal pain magnitude and unpleasantness ratings, and thermal pain thresholds. Alpha was set at 0.05.

## Results

In total, 2430 patients were called; 1527 could not be contacted or did not return our call, 185 declined, and 84 did not speak English and were excluded. A total of 634 patients consented to participate and completed the short survey, which included pain scores and the SF-MPQ-2. Of these patients, 367 at least partially completed the long survey and 202 patients completed a set of four psychophysical assessments.

Patient demographics and CPSP patient characteristics are reported in Tables 1 and 2, respectively. Prior to their procedure, the majority of patients (*N* = 367; 57.4%) reported experiencing preoperative chronic pain. The mean postsurgery assessment time for the cohort was 41.46 ± 25.10 months. At this time, 27.8% (*N* = 176) of all patients reported CPSP at the midline sternotomy site; 36 (5.68) others reported chronic pain at a site other than the midline sternotomy.

**Table 1.** Overall patient demographics.^a^

Characteristic	Total (*N* = 634)
Age (years), mean (SD)	65.92 (12.56)
Sex, *N* (%)	
Male	440 (69.50)
Female	193 (30.50)
BMI, mean (SD)	28.15 (5.25)
Previous chronic pain, *N* (%)	
Yes	367 (58.35)
No	262 (41.65)
First ever surgical procedure, *N* (%)	
Yes	191 (30.13)
No	443 (69.87)
Surgery post-CABG, *N* (%)	
Yes	127 (20.03)
No	507 (79.97)
Time from surgery to study consent (months), mean (SD)	41.46 (25.10)

^a^Percentages are calculated out of the number of valid responses for each specific variable and not out of the total sample (*N* = 634) because there are occasional missing data.

CABG(S) = coronary artery bypass graft surgery.

**Table 2.** Short survey post–cardiac surgery pain characteristics.^a^

Body part	Number of patients, *N* (%)	Pain intensity 0–10, mean (SD)
Chest pain from cardiac surgery	176 (27.8)	4.10 (2.29)
Mild pain	82 (12.93)	
Moderate pain	63 (9.94)	
Severe pain	31 (4.89)	
Other chronic pain only	36 (5.68)	5.22 (2.78)
Mild pain	11 (1.74)	
Moderate pain	14 (2.21)	
Severe pain	11 (1.74)	

^a^Percentages are calculated out of the number of valid responses for each specific variable and not out of the total sample (*N* = 634) because there are occasional missing data.

### Short survey

Of patients in the CPSP group, 46.6% reported low/mild pain (1–3 on the NRS_0–10_ scale), 35.8% reported moderate pain (4–6 on the NRS_0–10_ scale), and 17.6% reported severe pain (7–10 on the NRS_0–10_ scale). No sex differences in the development of CPSP were found. Results of the multivariable multinomial regression model showed that patients reporting moderate or severe CPSP were younger (*P* < 0.05), had a higher BMI (*P* < 0.001), and had a shorter postoperative follow-up time (*P* < 0.001) compared to those without CPSP (Table 3).

Of the 258 patients who had surgery to harvest the saphenous vein, 38 (14.7%) developed chronic leg pain. Of these 38 patients, 24 (63.2%) also developed midline sternotomy CPSP. Furthermore, patients reporting previous chronic pain elsewhere in the body were more likely to have CPSP (*P* = 0.006), with 52.3% of patients with CPSP reporting a prior history of chronic pain compared to just 37.5% for those who did not develop CPSP. This association was no longer significant in the multivariable multinomial regression model, however.

### Long survey

Age (*P* < 0.001), but not sex (*P* > 0.05), was significantly associated with CPSP status such that patients with CPSP were younger than those without CPSP. Unlike in the short survey, there were no significant differences in BMI with no CPSP as the reference category (*P* > 0.05). Among the psychological variables, results of the multivariable multinomial regression model showed that anxiety sensitivity was significantly higher among patients with moderate pain (*P* = 0.011); patients with severe CPSP had significantly higher levels of pain catastrophizing (*P* = 0.007) and patients with moderate or severe CPSP had significantly higher levels of somatization (*P* ≤ 0.001) compared to those without CPSP (Table 4).

### Psychophysical assessment

Generally, patients with CPSP (59 of the 202 participants) demonstrated significant differences on several noxious cold and heat assays but not on nonnoxious warm and cool psychophysical assays (Table 5). Results of individual tests are shown in Table 5 but only pain-related variables were included in the multivariable multinomial regression model. Results showed that patients with mild and moderate CPSP had significantly higher pain magnitude ratings 30 s postwithdrawal from the cold pressor test (*P* < 0.05). There were also significant differences in the thermal pain detection thresholds such that patients with pain had lower heat and cold pain detection thresholds at the forearm (*P* < 0.05), thus showing cold allodynia and heat hyperalgesia (Table 5).

**Table 3.** Short survey comparison of patients experiencing pain to those not experiencing pain.^a^

Characteristic	No CPSP (*N* = 453)	Mild CPSP (*N* = 82)	Moderate CPSP (*N* = 63)	Severe CPSP (*N* = 31)	*P* value (univariate analysis)	*P* value (multivariate analysis; no CPSP as reference category)
Age (years),					0.008	0.001 (moderate CPSP)*
mean (SD)	66.68 (12.63)	66.32 (11.74)	61.32 (13.87)	63.03 (8.18)		0.027 (severe CPSP)*
Sex					0.258	All *P*s > 0.05
Male	316 (69.76)	62 (75.61)	42 (66.66)	17 (56.66)		
Female	137 (30.24)	20 (24.39)	21 (33.33)	13 (43.33)		
BMI (kg/m^2^),					0.191	<0.001 (moderate and severe CPSP)*
mean (SD)	27.99 (5.22)	27.95 (4.90)	29.12 (6.02)	29.56 (5.02)		
Previous chronic pain					0.006*	
Yes	168 (37.50)	40 (48.78)	33 (52.38)	19 (61.29)		
No	280 (62.50)	42 (51.22)	30 (47.62)	12 (38.71)		
Time from surgery to study consent (months),					<0.001*	<0.001 (moderate and severe CPSP)
mean (SD)	46.64 (21.03)	45.66 (36.67)	13.11 (1.60)	13.48 (1.38)		
SF-MPQ-2						
Continuous, mean (SD)	0.70 (1.31)	0.89 (1.54)	0.72 (1.17)	1.13 (1.66)	0.274	
Intermittent, mean (SD)	0.42 (1.11)	0.47 (1.06)	0.50 (1.22)	0.93 (1.60)	0.112	
Neuropathic, mean (SD)	0.54 (1.07)	0.68 (1.16)	0.84 (1.29)	0.70 (1.17)	0.179	
Affective, mean (SD)	0.47 (1.14)	0.63 (1.20)	0.85 (1.41)	0.87 (1.96)	0.048*	
Overall scale, mean (SD)	0.54 (0.96)	0.67 (1.05)	0.72 (1.03)	0.91 (1.39)	0.114	

^a^All variables tested in the univariate models (except SF-MPQ-2 subscales) were entered in a backward selection multinomial regression model (except age and sex, which were forced into the model) with no CPSP as the reference category. Variables with no *P* values were not retained in the final model. Percentages are calculated out of the number of valid responses for each specific variable and not out of the total sample (*N* = 634) because there are occasional missing data.

*P values < 0.05 considered significant.

CPSP = chronic postsurgical pain; BMI = body mass index; SF-MPQ-2 = McGill Pain Questionnaire–Short Form–2.

**Table 4.** Long survey comparison of patients experiencing pain to those not experiencing pain.^a^

Characteristic	No CPSP (*N* = 267)	Mild CPSP (*N* = 52)	Moderate CPSP (*N* = 31)	Severe CPSP (*N* = 15)	*P* value (univariate analysis)	*P* value (multivariate analysis; no CPSP as reference category)
Age (years),					<0.001*	All *P*s < 0.01*
mean (SD)	64.32 (11.07)	59.46 (10.33)	57.26 (14.30)	58.20 (10.36)		
Sex					0.083	>0.05
Male	206	41	20	8		
Female	61	11	11	7		
BMI (kg/m^2^),					0.330	
mean (SD)	27.97 (4.92)	29.33 (5.59)	27.95 (4.71)	28.74 (5.57)		
Previous chronic pain					0.766	
Yes	101	25	14	9		
No	166	27	17	6		
Time from surgery to study consent (months),					0.003*	0.003 (mild CPSP)*
mean (SD)	43.25 (24.50)	30.38 (19.56)	35.40 (26.21)	42.01 (25.29)		
ASI-3						
Physical, mean (SD)	4.64 (4.56)	5.63 (4.67)	6.91 (5.73)	6.00 (5.62)		
Cognitive, mean (SD)	2.80 (3.94)	2.90 (3.78)	4.52 (5.50)	2.29 (3.02)		
Social, mean (SD)	4.86 (4.54)	6.92 (5.88)	6.84 (5.65)	4.50 (4.52)		
Total, mean (SD)	12.30 (11.07)	15.46 (12.96)	18.27 (15.00)	12.79 (11.55)	0.027*	0.011 (moderate CPSP)*
HADS						
Depression, mean (SD)	3.10 (3.02)	3.42 (3.30)	3.26 (3.67)	4.01 (3.62)	0.668	
Anxiety, mean (SD)	3.99 (3.20)	4.85 (3.01)	5.84 (3.62)	5.80 (4.25)	0.004*	
PASS-20						
Avoidance, mean (SD)	5.60 (5.30)	7.04 (5.47)	6.29 (6.44)	11.00 (7.50)		
Fear, mean (SD)	2.47 (3.92)	3.13 (4.28)	4.85 (6.28)	5.87 (6.89)		
Cognitive, mean (SD)	4.74 (5.44)	6.06 (5.51)	7.64 (7.05)	11.60 (7.85)		
Physiological anxiety, mean (SD)	1.88 (3.13)	2.48 (3.78)	4.64 (5.57)	4.97 (4.04)		
Total, mean (SD)	14.69 (15.71)	18.77 (16.74)	23.41 (22.47)	33.43 (23.68)	<0.001*	
SPTS						
Total, mean (SD)	19.33 (6.68)	20.33 (6.86)	22.06 (9.92)	28.20 (12.87)	<0.001*	
PCL-C						
Re-experiencing, mean (SD)	6.82 (2.59)	8.04 (3.34)	8.68 (4.17)	9.00 (4.11)		
Avoidance, mean (SD)	2.89 (1.53)	3.10 (1.82)	3.52 (1.91)	3.80 (2.15)		
Emotional numbing, mean (SD)	7.21 (2.92)	7.54 (3.20)	8.13 (4.48)	9.20 (4.11)		
Hyperarousal, mean (SD)	8.14 (3.29)	8.68 (3.00)	9.68 (4.57)	11.07 (4.17)		
Total, mean (SD)	25.06 (8.87)	27.36 (9.21)	30.00 (13.34)	33.07 (12.89)	0.001*	
PCS total						
Helplessness	2.86 (3.99)	3.60 (3.71)	4.53 (5.53)	7.47 (6.45)		
Rumination	3.72 (4.01)	3.62 (3.52)	4.94 (3.81)	7.47 (5.19)		
Magnification	1.53 (1.96)	2.10 (2.37)	2.71 (2.90)	3.53 (3.29)		
Total, mean (SD)	8.11 (9.14)	9.31 (8.70)	12.17 (11.11)	18.47 (13.57)	<0.001*	0.007 (severe CPSP)*
MAAS, mean (SD)	4.91 (0.77)	4.72 (0.81)	4.71 (0.72)	4.79 (0.67)	0.256	
SCL-90-R-Somatization,						
mean (SD)	6.04 (5.92)	8.03 (6.63)	11.96 (10.57)	14.60 (11.79)	<0.001*	<0.001 (moderate CPSP)*
						0.001 (severe CPSP)*

^a^All variables tested in the univariate models were entered in a backward selection multinomial regression model (except age and sex which were forced into the model) with no CPSP as the reference category. Variables with no *P* values were not retained in the final model. Percentages are calculated out of the number of valid responses for each specific variable and not out of the total sample (*N* = 634) because there are occasional missing data.

*P values < 0.05 considered significant.

CPSP = chronic postsurgical pain; BMI = body mass index; ASI-3 = Anxiety Sensitivity Index; HADS = Hospital Anxiety and Depression Scale; PASS-20 = Pain Anxiety Symptoms Scale–20; SPTS = Sensitivity to Pain Traumatization Scale; PCL-C = Posttraumatic Stress Disorder Checklist–Civilian Version; PCS = Pain Catastrophizing Scale; MAAS = Mindful Attention Awareness Scale; SCL = Symptom Checklist.

**Table 5.** Psychophysical assessments comparison of patients experiencing pain to those not experiencing pain.^a^

Characteristic	No CPSP (*N* = 143)	Mild CPSP (*N* = 25)	Moderate CPSP (*N* = 21)	Severe CPSP (*N* = 13)	*P* value (univariate analysis)	*P* value (multivariate analysis; no CPSP as reference category)
Age (years),					0.029	
mean (SD)	65.97 (11.49)	61.00 (10.37)	59.19 (13.70)	63.31 (10.52)		
Sex					0.270	
Male	115 (80.42)	22 (88.00)	16 (76.19)	8 (61.54)		
Female	28 (19.58)	3 (12.00)	5 (23.81)	5 (38.46)		
BMI (kg/m^2^),						
mean (SD)	27.77 (4.62)	27.24 (3.52)	28.32 (5.51)	28.67 (6.02)		
Previous chronic pain					0.128	
Yes	56 (39.16)	15 (60.00)	10 (47.62)	8 (70.54)		
No	87 (60.84)	10 (40.00)	11 (52.38)	5 (29.46)		
Mechanical pressure pain threshold						
Sternum force (kg), mean (SD)	7.98 (4.07)	7.92 (5.03)	6.15 (3.73)	5.97 (4.79)	0.131	
Right forearm force (kg), mean (SD)	9.48 (4.70)	10.15 (6.00)	8.67 (4.98)	7.63 (6.04)	0.448	
Scar saphenous vein graft force (kg), mean (SD)	6.59 (2.84)	8.67 (4.63)	4.25 (1.06)	7.43 (8.06)	0.270	
Left forearm force (kg), mean (SD)	9.42 (4.56)	9.67 (5.44)	8.43 (4.75)	7.50 (5.20)	0.452	
Mid-sternal scar NRS, mean (SD)	2.75 (1.87)	2.84 (1.67)	3.41 (2.42)	3.23 (1.68)	0.446^b^	
Right forearm NRS, mean (SD)	2.62 (1.84)	2.72 (1.77)	3.17 (2.31)	2.54 (1.38)	0.639	
Scar saphenous vein graft NRS, mean (SD)	3.01 (1.80)	3.08 (2.05)	2.00 (1.41)	3.00 (1.22)	0.890	
Left forearm NRS, mean (SD)	2.64 (1.81)	2.56 (1.48)	3.19 (2.27)	2.50 (1.57)	0.588	
Vasoconstrictor inspiratory gasp						
Baseline 1 systolic, mean (SD)	132.66 (21.33)	124.36 (10.52)	123.43 (18.11)	130.23 (15.80)	0.0380	
Baseline 2 systolic, mean (SD)	129.51 (19.83)	122.60 (11.24)	121.62 (17.14)	127.69 (17.23)	0.143395	
1-min postgasp systolic, mean (SD)	126.62 (19.24)	122.76 (10.27)	120.14 (15.59)	124.46 (14.78)	0.380	
Baseline 1 diastolic, mean (SD)	76.80 (10.21)	77.28 (6.89)	73.71 (8.15)	77.54 (7.07)	0.523	
Baseline 2 diastolic, mean (SD)	75.56 (9.52)	76.08 (6.49)	72.95 (7.68)	75.62 (8.16)	0.623	
1-min postgasp diastolic, mean (SD)	75.54 (8.85)	76.08 (6.87)	73.38 (7.32)	75.62 (7.66)	0.701	
Baseline 1 arterial blood pressure, mean (SD)	95.42 (12.49)	92.97 (7.57)	90.29 (10.72)	95.10 (9.21)	0.255	
Baseline 2 arterial blood pressure, mean (SD)	93.54 (11.45)	91.59 (7.49)	89.17 (10.51)	92.97 (10.64)	0.350	
1-min postgasp arterial blood presure, mean (SD)	92.57 (11.10)	91.64 (7.28)	88.97 (9.57)	91.90 (9.58)	0.531	
Baseline 1 pulse rate (beats per minute), mean (SD)	68.06 (12.46)	68.60 (10.98)	65.14 (9.79)	70.54 (9.16)	0.598	
Baseline 2 pulse rate (beats per minute), mean (SD)	67.77 (12.30)	67.12 (10.58)	64.38 (10.11)	69.92 (9.05)	0.544	
1-min postgasp pulse rate (beat per minute), Mean (SD)	67.39 (12.08)	68.40 (10.24)	64.81 (10.73)	70.00 (9.74)	0.598	
1-min postgasp spontaneous pain rating, mean (SD)	0.00 (0.00)	0.16 (0.62)	0.24 (0.77)	0.92 (1.98)	<0.001	
Cold pressor pain test						
Withdrawal time (s), mean (SD)	35.43 (47.15)	28.78 (37.02)	39.57 (59.24)	14.44 (5.64)	0.409	
30-s postwithdrawal pain magnitude rating, mean (SD)	0.64 (1.44)	1.46 (2.15)	2.57 (2.23)	0.64 (0.81)	<0.001	0.025 (mild CPSP)^b^ <0.001 (moderate CPSP)
30-s postwithdrawal pain unpleasantness rating, mean (SD)	0.67 (1.47)	1.42 (2.47)	2.52 (2.14)	0.36 (0.68)	<0.001^b^	
Thermal pain and sensory thresholds (units)						
WDT chest mean, mean (SD)	40.94 (4.03)	39.97 (3.87)	40.81 (4.31)	42.39 (4.71)	0.443	
CDT chest mean, mean (SD)	24.55 (6.12)	25.39 (5.01)	24.43 (6.00)	21.25 (9.42)	0.326	
HPT chest mean, mean (SD)	47.43 (4.47)	45.94 (3.16)	46.10 (3.43)	48.39 (1.81)	0.185	All *P*s > 0.05^b^
CPT chest mean, mean (SD)	3.84 (7.27)	7.10 (9.26)	4.77 (7.31)	0.73 (2.23)	0.095^b^	
WDT forearm mean, mean (SD)	38.98 (4.09)	37.97 (3.38)	38.21 (4.96)	38.98 (3.37)	0.636	
CDT forearm mean, mean (SD)	25.88 (5.09)	26.55 (4.00)	26.20 (3.09)	24.21 (4.67)	0.597	
HPT forearm mean, mean (SD)	46.52 (4.77)	45.00 (4.13)	45.17 (2.83)	45.70 (2.84)	0.310	All *P*s < 0.001^b^
CPT forearm mean, mean (SD)	5.07 (7.52)	7.14 (8.93)	9.74 (8.81)	5.61 (5.25)	0.071	0.015 (moderate CPSP)^b^

^a^Percentages are calculated out of the number of valid responses for each specific variable and not out of the total sample (*N* = 634) because there are occasional missing data. Items with *P* values indicate that they were retained in the final model.

^b^Variables examined in the multivariable multinomial regression model using a backward selection process.

*P values < 0.05 considered significant.

CPSP = chronic postsurgical pain; BMI = body mass index; NRS = numeric rating scale; WDT = warm detection threshold; CDT = cold detection threshold; HPT = heat pain threshold; CPT = cold pain threshold.

## Discussion

This is the largest postsurgical cohort study to date that reports on the pain and psychological phenotypes of patients who developed CPSP following cardiac surgery, in contrast to those not developing CPSP after the same surgery. In keeping with previous studies demonstrating CPSP rates ranging from 11% to 40% after cardiac surgery, we report that 27.7% of the patients studied in the present cohort developed such chronic pain.^8^

Of the 176 patients who reported CPSP, 46.6% reported low to mild pain, 35.7% reported clinically relevant moderate pain, and 17.6% reported clinically relevant severe pain in the operated field and the nearby region of the chest. Yet only approximately 6% of the cohort developed chronic pain following a concurrent surgery to harvest the saphenous vein. It is possible that the lower incidence of chronic postsurgical pain in the calf scar is due to the smaller extent of tissue and nerve injury or the use of a pre-emptive peri-operative use of local anesthesia. It is also possible that the use of special spanners to enable access to the heart for the duration of the surgery caused compression of intercostal nerves and added causes for increased rates of CPSP compared to calf surgery.

A majority of patients (63.2%) who developed chronic pain in the calf also developed sternotomy CPSP. Likewise, many of those with CPSP reported a prior history of chronic pain elsewhere in the body. These associations may suggest that chronic pain is an innate trait, controlled by the same genetic variants that express their potential whenever there is surgery. These findings are also compatible with the strong tendency to develop the same chronic pain if amputated twice in life, either concurrently or delayed or years apart.

### Psychological risk

In accordance with previous literature, patients presenting for surgery with prior chronic pain were more likely to develop CPSP.^17,18^ Depression and emotional numbing did not show a significant difference in reporting between the two groups unlike previous work.^13^ However, our study is congruent with previous work demonstrating that the transition to CPSP is associated with pain rumination,^5,19,20^ the tendency to catastrophize,^5,19,20^ pain magnification,^5,19,20^ functional disability,^5^ fear of pain and anxiety,^5,21^ and younger age.^5,22–24^

Based on existing validated models of chronic pain and disability (e.g., the diathesis-stress model of chronic pain),^25^ patients with CPSP are at risk of psychological diathesis from sensitivity to bodily sensations^26^ and anxiety sensitivity.^27^ With regards to the cognitive–behavioral fear-avoidance model, responses of patients with CPSP to psychological questionnaires indicate a greater likelihood of being fearful and avoidant of pain.^28,29^ Finally, this patient population reports increased pain catastrophizing compared to patients without chronic pain. Such individuals are likely to engage in catastrophic thinking if they are genetically predisposed to respond fearfully to pain and if they show signs of posttraumatic stress.^30^ Recent studies highlight the importance of assessing posttraumatic stress symptoms and their relevance to pain chronicity in patients undergoing major surgery.^13,31^ For these patients, when pain is perceived as a threat, a vicious cycle is initiated in which pain-related catastrophizing and anxiety dominate the patient’s experience. This leads to somatic hypervigilance, activity avoidance, and disability, which in turn feed back into the pain experience to fuel pain-related fears.^6^ Alternatively, it is possible that the same genes that predispose patients to transition to chronic pain due to a psychological vulnerability such as depression, anxiety, and catastrophizing may have a pleiotropic role in peripheral and/or central pain pathways. This could predispose patients to develop abnormal hyperexcitability in injured primary afferents and/or in central nerve system (CNS) networks that abnormally process afferent nociceptive inputs. An example of such a gene is *COMT*, polymorphisms in which have been associated with catastrophizing, anxiety, and fear (by operating in the CNS), and the same polymorphisms concurrently affect breakdown of adrenaline and noradrenaline in sympathetic efferents in injured nerve-end neuromas,^32^ where they may mediate sympathetically maintained pain.

### Psychophysical testing

Though patients with CPSP developed cold and hot allodynia in the forearms bilaterally and cold hyperalgesia in the arm and hand, no such abnormalities were detected in the chest. Regrettably, we did not use the quantitative sensory testing (QST) assay to test for these sensory abnormalities at the calf. However, in contrast to abnormalities at the noxious intensity range, patients with CPSP did not develop cool or warm hyperesthesia; that is, there were no differences between the groups with regards to detection thresholds of nonpainful sensations of cool or warm in the forearms.

Using mechanical algometry, we found that having CPSP was associated with a trend for mechanical allodynia in the sternum. Because patients were instructed to press a button to stop the further increase in stimulus intensity beyond the pain threshold, we do not know whether they also developed mechanical hyperalgesia. Not withstanding, based on the thermal sensory abnormalities discovered in this study, we can conclude that spontaneous CPSP at the field of sternotomy and cardiac surgery involves long-lasting sensitization of pain (but not of nonnoxious) pathways in the CNS to natural stimuli applied both intra- and extraterritorially. Sensory abnormalities within the region innervated by nerves injured during surgery are defined here as intraterritorial abnormalities, whereas extraterritorial abnormalities appear outside the field innervated by nerves injured during the surgical process.

One potential explanation is that signals stemming from injury may reach the somata of neighboring intact primary afferents to trigger long-term changes in the expression profile of genes encoding proteins engaged in their excitability (e.g., genes for voltage-gated ion channel components). However, evidence supports an alternative explanation termed *central sensitization*, whereby there are changes to the processing of sensory nociceptive CNS inputs, rather than changes in the periphery.^33^ Under normal conditions, many projection neurons in the spinal dorsal horn that are driven by nociceptive input also receive converging nociceptive and nonnociceptive inputs from receptive fields innervated by neighboring nerves. When activated by natural stimuli, these convergent afferent inputs from surrounding receptive fields are normally inhibited and unable to effectively drive those projection neurons. Thus, stimulation of the forearms and hands cannot normally activate CNS projection neurons that receive their main nociceptive input from the sternal and cardiac afferents and vice versa. This is at the heart of the high resolution of nociceptive and nonnociceptive somatotopic maps that process sensory inputs from all modalities. But after peripheral nerve injury (as occurring in midsternotomy and cardiac surgery), there are long-lasting changes to spinal segmental, intersegmental, and inhibitory pathways descending from supraspinal structures in the CNS. When disinhibited, spinal projection neurons become hyperexcitable and respond more vigorously to afferent nociceptive and nonnociceptive inputs from the center of their receptive fields, causing allodynia, hyperalgesia, and spontaneous pain.

This study additionally found that CPSP is associated with significantly increased spontaneous pain, evoked by an inspiratory gasp. This maneuver is known to activate a transient increased sympathetic tone throughout the body. This increase in spontaneous pain can be explained by the documented upregulation of the expression of genes encoding components of adrenoreceptors and their shipment downstream by axonal transport and assembly in the peripheral ends of injured afferents. It is here that the upregulated adrenoreceptors are excited by circulating adrenaline and noradrenaline released from postganglionic sympathetic efferents terminating in the injured nerves.^34,35^ Another plausible explanation for the above finding could be that the increased pain is due to deep inspiration exacerbating their chest wall nociceptors rather than from sympathetic input or a combination of the two mechanisms.

Surprisingly, we found that patients with CPSP have a lower baseline systolic blood pressures compared to patients without CPSP, suggesting that CPSP is associated with a decreased sympathetic tone at rest. This finding is counterintuitive because the presence of inescapable and undertreated pain is known to be associated with stress, depression, and anxiety.^5,36^ We cannot explain this result.

Finally, we found an association between patients with obesity (BMI > 25) and CPSP. This confirms the findings of Bruce et al., who reported that patients who were overweight or obese at the time of cardiac surgery were more likely to report chronic pain.^37^ A higher BMI increases the technical difficulty of cardiac surgery and may expose such patients to prolonged retraction and a higher incidence of chronic pain. Further, we did not find between-sex differences in our study. Though chronic pain is reported to be more prevalent in females, studies of chronic pain after hernia surgery do not find an association between sex and chronic pain.^38–40^ Of note, Taillefer et al. did not find sex to be a predictor of chronic pain after cardiac surgery.^41^

These results highlight that there are distinct physiologic differences in patients who have developed CPSP and those that who have not. Because this was a cross-sectional study, we are unable to comment on temporal changes in psychophysical testing. Further prospective, longitudinal investigations should seek to delineate this time course and the critical time points that differ as patients develop CPSP whereas others do not.

### Phenomic aspects of CPSP

Because CPSP cannot be cured nor prevented and available mediations do not provide sufficient pain relief, there is a need for novel treatment targets, which are expected to originate from pharmacogenomics. This solution will become especially effective because current estimates suggest that heritability of chronic pain ranges from 30% to 70%.^5^,^7^ This optimistic value suggests that treatment solutions based on pharmacogenomic information could capture part of the trait variance that is controlled by genetic determinants. Elucidation of such genetic vulnerabilities could eventually explain the interindividual variability in the symptomatic repertoire of patients with CPSP and, based on their individual “genetic fingerprints,” offer precise medicine for individualized treatments. Though several reviews suggest that CPSP is a heritable trait,^42^–^44^ few studies have identified specific genes predisposing humans to CPSP. Even fewer genes have been found to predispose humans to the transition of acute to chronic pain after surgery, which could be targets for a preventive approach.

Several challenges and considerations exist in the path forward in identifying novel CPSP genes for pharmacogenetic therapy.

First, the simplistic phenomic approach of having/not having CPSP likely does not reflect the heterogeneous nature of traits contributing to the expression of CPSP. Thus, treatments based on genes found using the arch-phenome of having/not having CPSP are expected to be less effective than those tailored based on the repertoire of symptoms and signs displayed by an individual patient. To accomplish this goal would necessitate using those secondary traits as phenomes for genetic analysis, because each of these CPSP-related traits is arguably controlled by unique genes.

Second, using having/not having CPSP as the only phenome for genetic analysis might miss the detection of many genes, which could be identified if using more refined, mechanism-based intermediate phenomes.

Third, this raises the question of how to identify the best intermediate phenomes. In the present study, we addressed this issue by first using clinical parameters of CPSP to classify patients as having/not having CPSP and then using this grouping strategy to survey potential phenomes and intermediate phenomes that could be used for genetic association. However, the final test of how effective a CPSP-related phenotype is as a phenome can only be made if the genetic association analysis identifies genetic polymorphisms of relevance to CPSP and the effect size of carrying these polymorphisms can explain a sizable portion of the heritable, allelic risk for CPSP. Thus, to assess the effectiveness of a phenome necessitates having genotypic data of a sufficiently statistically powered cohort of patients with CPSP.

## Conclusion

Phenotyping pain and psychological questionnaires and psychophysical tests highlight key differences between patients who developed chronic pain after cardiac surgery and those who had the same surgery but did not develop such chronic pain. This is the first step in a study that aims at identifying a set of phenomes to be used in genetic association analysis of CPSP. Prospective longitudinal investigations are required to fully characterize the temporal aspects of these traits and identify genetic and environmental risk and protective factors underlying the transition from an acute pain to CPSP, its maintenance, and natural resolution in some patients.
